# Appalachian Regional Commission Recovery Ecosystem Background and Overview

**DOI:** 10.13023/jah.0203.03

**Published:** 2020-07-19

**Authors:** Kostas Skordas, Andrew Howard

**Affiliations:** M Urban/Regional Planning, MPA, Director, Research and Evaluation, Appalachian Regional Commission; Chief of Staff, Federal Co-Chair, Appalachian Regional Commission

**Keywords:** Appalachia, substance abuse, recovery, Appalachian Regional Commission, rural health

## Abstract

The Appalachian Regional Commission (ARC) has long addressed issues of substance abuse through funded community-based interventions, research, and sponsored conferences. Recently, the opioid crisis created a new urgency for cross-sector collaboration among various partners and funders dealing with this issue. This commentary provides an overview of recent efforts by the ARC to convene stakeholders to focus on assisting individuals with substance abuse disorder to access recovery services while pursuing education and training necessary to reenter the workforce and gain employment. Two papers have been prepared to describe this year-long effort. One paper focuses on the participatory methods used to gather ideas and turn them into regional intervention programs. The second paper describes and analyzes the wealth of ideas collected through the process. Both are intended to inform policymakers, researchers, and local leaders in ways to strengthen the substance abuse recovery ecosystem in their own communities.

The Appalachian Regional Commission (ARC) has long been involved in the issue of substance abuse. Across decades, the names of the substances regularly change, but the individual misery and population impacts seem no less disastrous over time. Through research studies, funded community-based intervention programs, sponsored conferences, and publications, substance-abuse disorder (SUD) prevention and treatment issues in the region have been extensively explored and described. One underlying theme that consistently emerges is that those affected by SUD are frequently removed from the region’s workforce with a profoundly negative impact on workforce participation rates and economic growth.

In the past few years new dollars began flowing from national and state sources as a result of the highly visible opioid crisis. A wide range of prevention, treatment, and recovery funding streams have been created. While appreciating the attention and new SUD resources, Appalachian leaders expressed concern about an ability to coordinate funds and to focus on an important goal—assisting individuals with SUD to access an organized set of recovery services while pursuing training and education necessary to reenter the workforce and gain employment. Experts and community leaders consistently state that having a job is pivotal to successfully maintaining recovery.

Appalachian Regional Commission leaders carefully considered what role the agency could most effectively play in to address this challenge. Building on decades of successful experience in engaging the empathy, energy, and expertise within communities, ARC pursued a process to define the elements needed to create community recovery ecosystems that could achieve the stated goal, then pursue targeted resources to assist communities to organize an appropriate mix of services and interorganizational agreements. This effort was titled Recovery to Work.

Importantly, ARC chose to listen first, then act on community ideas. The wisdom from the field was sought through state-based listening sessions. Findings from these meetings were reviewed by an advisory council who formed recommendations on how to organize and deploy ARC resources to spur development of local recovery ecosystems.

Two papers^1^,^2^ have been prepared to describe this year-long effort. Each describes an important aspect of the ARC Recovery Ecosystem approach. One paper focuses on the year-long participatory methods used to gather ideas and turn them into regional intervention programs. How to efficiently gather community input to ensure effective targeting of federal resources is an important lesson to share. The second paper describes and analyzes the wealth of ideas collected through the process. Little was found in the literature about recovery to work issues, so sharing the results of the ARC effort will represent new knowledge in the fields of workforce development and substance abuse services.

The first manuscript, Responding to Appalachian Voices: Steps in Developing a Regional Recovery to Work Initiative,^1^ describes the ARC sequential process to develop a recovery ecosystem model and create a new funding initiative. A mix of action research methods are reported. The paper demonstrates an efficient and effective process that listened first, then used community input to design resource allocations that support Recovery to Work. This paper will be of interest to policymakers faced with a similar challenge, that of conceptualizing and implementing new approaches to address old problems. The paper provides an example for sector leaders (e.g., state agencies, substance abuse treatment and recovery organizations, workforce development programs, and employer groups) about how to define and move on their common interests and missions.

The second manuscript, Listening to Voices in Appalachia: Gathering Wisdom from the Field to Develop Substance Abuse Recovery Ecosystems,^2^ reports new data collected directly from rural Appalachian communities. Data are presented from six listening sessions and organized using the elements of an ARC Recovery Ecosystem Model. The paper documents personal, organizational, and systems issues. The organization and attendance at listening sessions facilitated multi-sector discussion that identified interorganizational linkages and operational handoffs as critical for ecosystem’s success. This paper is the first introduction of the recovery ecosystem concept to the region, state agencies, and sector leaders. New secondary data about substance abuse disorders and workforce development in Appalachia is presented to encourage future research.
